# Supplementary material for: Determinants of anemia among pregnant women attending antenatal care in Horo Guduru Wollega Zone, West Ethiopia: Unmatched case-control study
Source: PLoS One. 2019 Oct 31;14(10):e0224514. doi: 10.1371/journal.pone.0224514 (PMC6822753; doi:10.1371/journal.pone.0224514)
Supplement: S1 File — (DOCX) [file pone.0224514.s001.docx]

**S1 File Table A: Maternal Obstetric factors of anemia among pregnant women who were attending ANC in the public health facilities of Horo Guduru Wollega Zone, West Ethiopia from September to October 25, 2017(n=573).**

| Variables | | **Frequencies(%)** | |
| --- | --- | --- | --- |
|  |  | **Cases** | **Controls** |
| Age of marriage | 15-19 | 100(53.2) | 194(51) |
|  | 20-24 | 86(45.5) | 173(45.4) |
|  | 25-29 | 3(1.6) | 14(3.7) |
| Menstrual bleeding in days | >8 | 46(24.1) | 32(8.4) |
|  | <8 | 145(76) | 350(91.6) |
| Heavy menstrual bleeding | Yes | 61(31.9) | 61(16) |
|  | No | 130(68.4) | 321(83.8) |
| Parity | Nulliparous | 53(28) | 132(34.5) |
|  | Primipara | 40(20.9) | 80(20.9) |
|  | Multipara | 45(23.7) | 92(24) |
|  | Grandpara | 53(28) | 78(20.4) |
| Birth interval in years | <2 | 26(13.7) | 21(5.5) |
|  | >2 | 117(61.1) | 271(71) |
| Antenatal care follow up | Yes | 147(77) | 350(91.6) |
|  | No | 44(23) | 32(8.4) |
| Bleeding | Yes | 22(11.6) | 13(3.4) |
|  | No | 169(88.5) | 369(96.6) |
| Nausea and vomiting | Yes | 63(33) | 89(23.3) |
|  | No | 128(67) | 293(76.7) |
| Trimester | First | 12(6.3) | 34(8.9) |
|  | Second | 57(29.8) | 107(28) |
|  | Third | 122(63.9) | 241(63) |

**S1 File Table B: Maternal dietary factors of anemia among pregnant women who were attending ANC in the selected public health facilities of Horo Guduru Wollega Zone, West Ethiopia from September 7 to October 25, 2017(n=573).**

| **Variables** | | **Frequencies (%)** | |
| --- | --- | --- | --- |
|  |  | **Cases** | **Controls** |
| Took iron | Yes | 104(54.5) | 273(71.5) |
|  | No | 87(45.8) | 109(28.5) |
| Taking iron supplement regularly | Yes | 63(60.6) | 239(87.5) |
|  | No | 41(39.4) | 34(12.4) |
| Time to take iron | 2 hours before meal | 5(4.8) | 15(5.5) |
|  | With meal | 46(44.2) | 80(29.3) |
|  | 2 hours after meal | 45(43.3) | 169(62) |
|  | Others | 8(7.6) | 9(3.3) |
| Eating raw meet | Yes | 55(28.8) | 104(27.2) |
|  | No | 136(71.2) | 278(72.8) |
| Eating Fish | Yes | 23(12.1) | 68(17.8) |
|  | No | 168(88) | 314(82.2) |
| Eating egg | Yes | 168(88) | 354(92.7) |
|  | No | 23(12) | 28(7.3) |
| Frequency of eating eggs | <3 per week | 19(11.2) | 92(26) |
|  | >3 per week | 150(88.8) | 262(74) |
| Frequency of eating vegetables | >3 per week | 56(29.3) | 175(45.8) |
|  | <3 per week | 135(70.7) | 207(54.2) |
| Frequency of eating fruits | >3 per week | 12(6.3) | 35(9.2) |
|  | <3 per week | 179(93.7) | 347(90.8) |
| Drinking tea | Yes | 147(77) | 312(81.7) |
|  | No | 44(23) | 70(18.3) |
| Frequency of drinking tea | 1 per day | 84(56.8) | 211(68) |
|  | 2-3 per day | 9(6.1) | 16(5.1) |
|  | 1-2 per week | 49(33.6) | 68(21.7) |
|  | >3per week | 5(3.4) | 16(5.1) |
| Drinking coffee | Yes | 159(83.2) | 316(83) |
|  | No | 32(16.8) | 66(17.2) |
| Time to take coffee | Before meal | 11(6.9) | 16(5) |
|  | With meal | 66(41.5) | 115(36.4) |
|  | After meal | 73(45.9) | 174(55) |
|  | Other | 9(5.6) | 12(3.4) |
| Frequency of eating cereals per week | Daily | 96(50.3) | 226(59.2) |
|  | Once | 27(14.2) | 45(11.7) |
|  | 2-3times | 54(28.4) | 86(22.5) |
|  | >3 times | 13(6.8) | 22(5.7) |
|  | 1 per month | 1(0.5) | 2(0.5) |
| Main staple foods | Teff | 130 (68) | 266 (69.6) |
|  | Barley | 27(14.2) | 62(16.2) |
|  | Wheat | 3(1.6) | 9(2.4) |
|  | Maize | 31(16.2) | 45(11.8) |
| Nutritional education | Yes | 121(63.4) | 274(71.7) |
|  | No | 70(36.6) | 108(28.2) |
| Mid-upper arm circumference | <23 | 96(50.3) | 58(15.2) |
|  | >23 | 95(49.7) | 324(84.8) |
| Daily dietary score | Low | 57(30) | 26(6.8) |
|  | Medium | 126(66) | 257(67.3) |
|  | High | 8(4.2) | 99(25.9) |
