# Supplementary material for: Determinants of anemia among pregnant women attending antenatal care in Horo Guduru Wollega Zone, West Ethiopia: Unmatched case-control study
Source: PLoS One. 2019 Oct 31;14(10):e0224514. doi: 10.1371/journal.pone.0224514 (PMC6822753; doi:10.1371/journal.pone.0224514)
Supplement: S2 File — (DOCX) [file pone.0224514.s002.docx]

## Questionnaires used to collect data from pregnant women

**Participant information sheet**

Good morning / Good afternoon and well come, my name is……………………………**.** I am a data collector for the study being conducted at this facility by Birhanu Daba, who is conducting study as partial fulfillment for the requirement of master’s degree in public health at Wollega University. I kindly ask you to give me your time to explain for you about the study and why you are being selected as the study participant.

The study will be conducted on determinants of anemia among pregnant women attending ANC in public health facilities of Horo Guduru Wollega zone. Knowing these factors has a great importance for governmental and non-governmental bodies to plan on intervention programs to reduce anemia. I will interview you using a questionnaire to be answered by you and I will fill the questionnaire that is helpful for the study within 20-30 minutes. So, I kindly request you to give me a few minutes for interview.

In addition to interview, small amount of blood and stool sample will be taken for laboratory test which is helpful for the study as well as for your benefit with minimal pain during blood draw for laboratory examination. If there is any finding from laboratory examination, you will be treated immediately at this facility. There is no any direct payment for participating in this study.

The information you will provide us will be confidential and it will not identify you in particular. The questionnaire will be coded to exclude showing name and no reference will be made that link participant to the research. Participation for this study is fully voluntary.

**Declaration of Informed Voluntary Consent**

I understood the purpose, procedures, risks and benefits, issues of confidentiality, the rights of participating and the contact address for any queries. Therefore, I declare my voluntary consent to participate in this study.

**Contact address:** Principal investigator: dababirhanu2@gmail.com or Mobile: 0910905865

**Thank You!**

## English Version Questionnaires

**Code ……… Date of data collection…………**

Table1.Questionnares on determinants of anemia among pregnant women

**I. Demographic and socioeconomic profile of study participants**

| 1 | How old are you? | | ------------------ |
| --- | --- | --- | --- |
| 2 | Where is your residence? | | 1. Urban  2. Rural |
| 3 | What is your current marital status? | | 1.Never married  2.Married  3.Widowed  4. Divorced |
| 4 | What is your religion? | | 1. Protestant  2. Orthodox  3.Muslim  4.Catholic  5.Other ( specify)-------------------- |
| 5 | What is your ethnicity? | | 1.Oromo  2.Amhara  4. Tigre  5.Other specify -------------------- |
| 6 | What is your family size? | | __________ |
| 7 | What is your occupation? | | 1.Government employee  2.Farmer  3.Merchant  4.Daily laborer  5.Other/specify------------------ |
| 8 | What is your husband’s occupation? | | 1.Government employee  2.Farmer  3.Merchant  4.Daily laborer  5.Other/ specify------------------ |
| 9 | What is your average monthly income in ETB? | | _________________ |
| 10 | What is your educational level? | | 1. No education  2.Primary schools  3.Secondary school  4.Above secondary school |
| **Π. Maternal Obstetric Factors** | | | |
| 11 | | If you were married, what was your age of marriage? | __________ |
| 12 | | Have you heavy menstrual bleeding during your menstrual period before the current pregnancy (using more than 7 pads per period? | 1.Yes  2.No |
| 13 | | Have you prolonged menstrual period (>8days)? | 1.Yes  2.No |
| 14 | | Is this the first pregnancy for you? | 1. yes  2. No |
| 15 | | If no to question number 14, how old were you when you first gave birth? | _______________ |
| 16 | | If you gave birth previously, what is your total number of pregnancies? | _______________ |
| 17 | | If no to question number 14, what is the total number of births you gave previously? | _______________ |
| 18 | | If no to question number 14, have you history of abortion before the current pregnancy? | 1.Yes  2.No |
| 19 | | If yes to question number 18, how many times? | _________________ |
| 20 | | If no to question number 14, what is the space between the current pregnancy and the last child | _______________ |
| 21 | | How many months are you with this pregnancy? | --------- |
| 22 | | How many times did you visit antenatal care service with this pregnancy? | ---------- |
| 23 | | At what month did you start to go for antenatal care service for the current pregnancy? | ----------- |
| 24 | | Have you ever used family planning methods? | 1. Yes  2. No |
| 25 | | If yes to question #24, what type of family planning? | 1.Implant  2. IUCD  3.Permanent  4. Pills  5. Depo-Provera  6. Others ( specify) |
| 26 | | Have you history of bleeding recently? | 1. Yes 2. No |
| 27 | | If yes to question #26, What type of bleeding? | 1. APH  2. Surgery  3. Trauma  4.Haemmorrhoid  5.Other ( specify)----------- |
| **III. Dietary Related Factors** | | | |
| 28 | | Did you take iron tablets during this pregnancy? | 1. Yes  2. No |
| 29 | | If Yes to question #28, how do you take? | 1. Daily  2.Not daily |
| 30 | | If yes to question #28, when do you take iron? | 1. 2 hours before meal  2. Immediately with meal  3. 2 hours after meal  4.Other(specify)-------------- |
| 31 | | How many times meals per day do you eat during the current pregnancy? | 1. One times  2. Two times  3. Three times  4.Four times  5. Five times |
| 32 | | Do you eat red meat? | 1.Yes  2.No |
| 33 | | If yes to question # 32, how many times do you eat red meat? | 1. Daily  2. Once per week  3. 2-3 per week  4. ≥ 4 per week  5. once per month  6.other ( specify)------------------ |
| 34 | | Do you eat fish? | 1.Yes  2.No |
| 35 | | If yes to question #34, how many times do you eat fish? | 1.Daily  2.once per week  3.2-3 per week  4. ≥ 4 per week  5.once per month  6.other specify |
| 36 | | Do you eat eggs? | 1.Yes  2.No |
| 37 | | If yes to question # 36, how many times do you eat eggs? | 1.Daily  2. Once per week  3. 2-3 per week  4. ≥ per week  5.Once per month  6.other specify |
| 38 | | Do you eat green leafy vegetables | 1.Yes  2.No |
| 39 | | If yes to question # 38, how many times do you eat green leafy vegetables (lettuce, spinach, cabbage, and broccoli)? | 1.Daily  2.once per week  3.2-3 per week  4. ≥ 4 per week  5.once per month  6.other specify |
| 40 | | Do you eat fruits (banana, mango, orange, papaya)? | 1.Yes  2.No |
| 41 | | If you eat fruits, how many times do you eat? | 1.Daily  2.once per week  3.2-3 per week  4. ≥ 4 per week  5.once per month  6.other specify |
| 42 | | Do you consume milk products (butter, cheese, and yogurt)? | 1. Yes  2. No |
| 43 | | If yes to question #42, how many times? | 1.Daily  2.once per week  3.2-3 per week  4. ≥ 4 per week  5.once per month  6.other specify |
| 44 | | If yes to question #28, do you take iron tablet with milk products? | 1. yes  2. No |
| 45 | | Do you drink tea? | 1. yes  2. No |
| 46 | | If yes to question # 45, how many times per day? | 1. one time  2. 2 -3 times  3. 1-2 per week  4. 3 or more per week |
| 47 | | Do you drink coffee? | 1.Yes  2. No |
| 48 | | If yes to question # 47, when do you drink coffee? | 1.2 hours before meals  2. With meals  3.2 hours after meals  4. Other (specify)________ |
| 49 | | Do you eat legumes? | 1.Yes  2.No |
| 50 | | If you eat legumes, how many times do you eat? | 1.Daily  2.once per week  3.2-3 per week  4. more than 3 per week  5.once per month  6. Other /specify………………… |
| 51 | | If yes to question # 49, what type of legumes or food made from legumes do you eat? | 1.Pease  2. Chickpeas  3. Inseed  4. Lentils  5. Beans  6. Niger seed  7. Sesame  8. Other (specify) |
| 52 | | Do you eat cereals or food made from cereals? | 1.Yes  2.No |
| 53 | | If yes to question # 52, How many times do you eat cereals? | 1. Daily  2. once per week  3.2-3 per week  4. ≥ 4 per week  5. once per month  6.Other ( specify)--------------------- |
| 54 | | If yes to question 52, what type of cereals do you eat? | 1. Sorghum  2. Barley  3. Wheat  4. Maize  5. Rice  6. Spaghetti  7. Teff  8. Other/specify…………………. |
| 55 | | What are your main staple foods? | 1. Teff 4. Maize  2. Barley 5. Sorghum  3. Wheat 6. Other/ specify……… |
| 56 | | Have you ever been exposed to nutritional education? | 1. Yes  2. No |
| **IV. Diseases Related Factors** | | | |
| 57 | | Have you ever been treated with malaria infection during this pregnancy? | 1. Yes  2. No |
| 58 | | Do you use bed ITN currently? | 1.Yes  2.No |
| 59 | | Do you wear shoes every day when you go out of house? | 1. Yes  2.No |
| 60 | | Have you experienced diarrhea in the last two weeks? | 1.Yes  2. No |
| 61 | | Have you any diseases currently? | 1.Yes  2. No |
| 62 | | If yes to question # 61, what type of diseases? | 1. Dyspepsia  2. Tb  3.HIV/AIDS  4.DM  5. Other specify---------- |
| **V. Hygiene and Sanitation Related Factors** | | | |
| 63 | | Do you use latrine? | 1. Yes  2.No |
| 64 | | If yes to question # 63, is there hand washing facility? | 1. Yes  2. No |
| 65 | | If no to question #63, Where do you use? | 1. Open field  2.Public latrine  3.Other specify--------------------- |
| 66 | | Where do you dispose solid waste? | 1.Open field  2. Open pit  3.Sanitary land fill  4.Other (specify) |
| 67 | | Where do you dispose sullage? | 1.Open field in the compound  2.Open pit  3. Closed pit  4.Other ( specify) |
| 68 | | What is the source of your drinking water? | 1. Tap water  2. protected spring/well  3. unprotected spring  4. River  5.Pond  6.Other ( specify)-------------- |
| 69 | | If your water source is other than tap water, do you treat it? | 1.Yes  2. No |
| 70 | | If yes to question # 69, by what method do you use? | 1. Chemical  2. Boiling  3. Filtering  4. Other (specify)------------ |
| 71 | | Do you wash your hand always before meal? | 1.Yes  2.No |

**VI. Dietary Diversity Score in 24 hour Recall for Pregnant Women**

| S. no | Minimum Dietary Diversity score for pregnant women food groups | Response |
| --- | --- | --- |
| 1 | Starchy staple foods(wheat, barley, maize, rice, potatoes, yams) | 1. Yes  2. No |
| 2 | Beas and Peas | 1. Yes  2. No |
| 3 | Nuts and seeds grains | 1. Yes  2. No |
| 4 | Dairy (milk and milk products) | 1. Yes  2. No |
| 5 | Flesh foods | 1. Yes  2. No |
| 6 | Eggs | 1. Yes  2. No |
| 7 | Vitamin A rich dark green leafy vegetables(Cabbage, spinach, broccoli) | 1. Yes  2. No |
| 8 | vitamin A –rich fruits(banana, apple, apricots) | 1. Yes  2. No |
| 9 | Non-green vegetables(sweet potato, carrots, lettuce) | 1. Yes  2. No |
| 10 | Other fruits(Orange, tomatoes, capsicum) | 1. Yes  2. No |

**VII. Laboratory Finding and Anthropometry**

| 1 | Maternal hemoglobin | …………g/dl |
| --- | --- | --- |
| 2 | Stool examination | 1. No ova parasite  2. Type of parasite seen…….  3. Other intestinal protozoa seen(specify)------------------ |
| 3 | Blood film | 1. No parasite  2. Parasite seen (specify)…… |
| 4 | VDRL test | 1.Yes  2. No |
| 5 | Maternal MUAC | -----------cm |
| 6 | Maternal height | __________ |

**THANK YOU!**

**Guca Eeyyama Hirmaattotaa**

Akkam bulte? Baga nagaan dhufte. Maqaankoo ------------------------------ jedhama.Ani ragaa sassaabaa qorannoo dhaabbata fayyaa kanatti Birhaanuu Dhaabaatiin barnoota digirii lammaffaa yuuniversiitii Wallaggaatti guuttachuuf taasisuuf hojjechaan jira.Kanaaf waa’ee qorannichaa fi maaliif hirmaattuu qorannichaa taate akka filatamte siif ibsuuf yerookee akka naaf kennitu kabajaan si gaafadha.

Qorannoon kun sababoota hir’ina dhiigaa dubartoota ulfaa hordoffii dahumsa duraa dhaabbilee fayyaa mootummaa Godina Horroo Guduruu Wallaggaatti taasisan irratti gaggeeffama. Sababoota kana beekuun qaamoleen mootummaa fi mit-mootummaa karoora ittisa hir’ina dhiigaa xiqqeessuu qabaachuuf faayidaa guddaa qaba. Unka gaafannootti fayyadamuun ergan sigaafadhee booda qorannichaaf kan fayyadu deebiikee unka gaafannoo irratti yeroo daqiiqaa 20-30 hin caalle keessatti nan guuta. Kanaaf yerookeerraa daqiiqaa muraasa akka naaf eeyyamtuuf kabajaan siin gaafadha.

Gaafannoo kanatti dabalataan, dhiiga xiqqoo fi bobbaan qorannoo laaboraatooriitif ta’u sirraa yommuu fudhatamu qorannichaafis ta’e siifis bu’aa kan qabu too ta’u, yeroo dhiigni qorannoodhaaf fudhatamu dhukkubbii xiqqoo dhiibbaa sirratti hin qabne sitti dhagahamuu ni danda’a. Qorannoo kanarraa dhibeen kan argamu yoo ta’e hatattamaan dhaabbatuma fayyaa kanatti yaaliin siif ni taasifama.Qorannoo kanarratti hirmaachuukeetiif kaffaltiin adddatti siif kaffalamu hin jiru.

Odeeffannoon ati nuuf kennitu icciitiidhaan Kan qabamuu fi siin adda baasee kan agarsiisu miti. Gaafannichi mallattoo icciitii /kooddii maqaa adda hin baafne kan qabuu fi agarsiiftuun hirmaattuu qorannichaa adda baasu hin jiru. Hirmaannaan qorannoo kanaa guutummaatti fedhii irratti kan hundaahedha.

**Ibsa eeyyama hirmaannaa fedhii irratti hundaa’e**

Ani sababa, tartiiba, miidhaa fi faayidaa, dhimma icciitii fi mirga qorannicha irratti hirmaachuu fi gaaffii kamiifuu qaaman dubbisu hubadheera.Kanaaf jechaan eeyyama fedhiidhaan qorannicha irratti hirmaachuukoo nan beeksisa.

**Bakka quunnamtii: Lakkoofsa bilbilaa: 0910905865**

**Gmail: dababirhanu2@gmail.com GALATOOMAA!**

## Gaaffaannoo Afaan Oromiffaa

**Kooddii Hirmaattuu………… Guyyaa ragaan itti funaaname…………..**

Gabatee 2. Gaafannoo sababoota dhibee hanqina dhiigaa dubartoota ulfaarratti fidanii

I. Faayela Dimoogiraafii, Qabeenyaa fi Hawwaasummaa

| 1 | Umuriinkee meeqaa? | _______________ | |
| --- | --- | --- | --- |
| 2 | Bakki jireenyakee eessaa? | 1. Magaala  2. Baadiyaa | |
| 3 | Haalli gaa’elakee Yeroo ammaa maal fakkaata? | 1. Kan hin heerumne  2. Kan heerumte  3.Abbaan manaa kan du’e  4. kan wal-hiikan | |
| 4 | Amantaan kee maalii? | 1. Pirootestaantii  2. Ortodoksii  3.Muusiliima  4.Kaatolikii  5.Kan biroo( ibsi)-------------------- | |
| 5 | Sabummaankee maalii? | 1 Oromoo  2.Amaara  4. Tigiree  5.Kan biroo(ibsi) -------------------- | |
| 6 | Baay’inni maatiikee meeqaa?? | _________ | |
| 7 | Hojiinkee maalii? | 1.Hojjettuu mootummaa  2.Qotee bulaa  3.Daldaaltuu  4.Hojii guyyaa  5.Kan biroo(ibsi)------------------ | |
| 8 | Hojiin abbaa manaa kee maalii? |  | |
| 9 | Galiin ji’aakee jiddu-galeessaan Qarshii meeqaa? | ----------------- | |
| 10 | Sadarkaan barnootakee maalii? | 1.kan hin baranne  2.Sadarkaa tokkoffaa(1-8)  3.Sadarkaa lammaffaa(9-10)  4. Sadarkaa lammaffaan ol(>10) | |
| **Π. Sababoota hir’ina dhiigaa haadha waliin wal –qabatan** | | | |
| 11 | Yoo gaa’ela dhaabbatteetta ta’e, umurii meeqatti gaa’ela dhaabbattee? | | ____ |
| 12 | Yeroo xuriin sitti dhufu dhiigni ni baay’ataa ulfa kana dura (moodesii 7 ol ni jijjiirrattaa guyyaa tokkotti? | | 1.Eyyee  2.Lakki |
| 13 | Xuriin guyyoota dheeraa (>8 guyyaa) sirra turaa? | | 1.Eyyee  2.Lakki |
| 14 | Ulfi ammaa kun jalqabakeeti? | | 1.Eyyee  2.Lakki |
| 15 | Jalqaba Yeroo deesse umuriinkee meeqa? | | ____________ |
| 16 | Ulfi kun yoo jalqabakee miti ta’e, Yeroo meeqa garaatti sihafeera? | | ------------- |
| 17 | Yoo deebiin gaaffii # 14 lakkii ta’e, hanga ammaatti daa’imma meeqa deesseettaa? | | ------------ |
| 18 | Yoo deebiin gaaffii # 14 lakkii ta’e, ulfikee addaan citee yookaan sirraa bahee ni beekaa? | | -------------- |
| 19 | Ulfi sirraa ba’ee kan beeku yoo jiraate yeroo meeqa? | | ------------------- |
| 20 | Yoo deebiin gaaffii # 14 lakkii ta’e, turtiin ulfa kan ammaa fi dahumsa isa darbe jidduu jiru meeqa? | | _________ |
| 21 | Erga garaatti si hafee ji’a meeqaffaadhaa? | | --------- |
| 22 | Ulfa ammaa kanaaf Yeroo meeqa hordoffii gooteettaa? | | ------------ |
| 23 | Ulfa Yeroo ammaa kanaaf ji’a meeqaffaa keetti hordoffii dahumsa duraa jalqabdee? | | ----------- |
| 24 | Karoora maatiitti fayyadamtee beektaa? | | 1. Eyyee  2. Lakki |
| 25 | Deebiinkee gaaffii #24 eyyee yoo ta’e, gosa kam? | | 1.Impilaantii  2. Luuppii  3.Dhaabbataa  4. Piilsii  5. Marfee ji’a sadii  6. Kan biroo( ibsi) |
| 26 | Yeroo dhiyootti dhigni si dhiigee ni beekaa? | | 1. Eyyee 2. Lakki |
| 27 | Yoo deebiin #26 eyyee ta’e, gosa dhiigaa kam? | | 1. Dhiiga yeroo ulfaa  2. Dhiiga baqaqsanii yaaluu  3. Miidhaa balaa tasaa  4.Kintaarotii  5.Kan biroo( ibsi)----------- |
| **III. Sababoota Haala Nyaataa Waliin Wal-qabatan** | | | |
| 28 | Taabileetii ayireenii ulfa ammaa kanaaf fudhatteettaa? | 1. Eyyee  2. Lakki | |
| 29 | Yoo deebiin gaaffii #28 eyyee ta’e , yeroo kam fudhatta? | 1. Guyyaa guyyaatti  2.Guyyaa guyyaadhaan miti | |
| 30 | Yoo deebiin # 28 eyyee ta’e, Yeroo kam taabileetii Ayireenii fudhattaa? | 1. Sa’aatii 2 nyaataa dura  2. Nyaata waliin  3. Sa’aa 2 nyaata booda  4. Kan biroo(ibsi)------------ | |
| 31 | Ulfa Yeroo ammaarratti nyaata guyyaatti yeroo meeqa nyaattaa? | 1. Yeroo tokko  2. Yeroo lama  3. Yeroo sadii  4.Yeroo afur  5. Yeroo shan | |
| 32 | Foon diimaa ni nyaattaa? | 1.Eyyee  2.Lakki | |
| 33 | Yoo deebiin gaafffii # 32 eyyee ta’e, foon diimaa Yeroo hammamiitti nyaatta? | 1. Guyyaa guyyaatti  2. Torbeetti Yeroo tokko  3. Torbeetti Yeroo 2-3  4. Torbeetti Yeroo 4 fi isaa ol  5. Ji’atti Yeroo tokko  6. Kan biroo ( ibsi)------------------ | |
| 34 | Foon qurxummii ni nyaattaa? | 1.Eyyee  2.Lakki | |
| 35 | Yoo deebiin gaaffii # 34 eyyee yoo ta’e, foon qurxummii yeroo meeqa nyaattaa? | 1.Guyyaa guyyaatti  2.Torbeetti Yeroo tokko  3.Torbeetti Yeroo 2-3tti  4. Torbeetti Yeroo 4 fi isaa ol  5.Ji’atti Yeroo tokko  6. Kan biroo (ibsi)---------------- | |
| 36 | Hanqaaquu ni nyaattaa ? | 1.Eyyee  2. Lakki | |
| 37 | Yoo deebiin gaaffii 36 eyyee ta’e, hanqaaquu Yeroo meeqa nyaattaa? | 1.Guyyaa guyyaatti  2.Torbeetti altokko  3.Torbeettti Yeroo 2-3tti  4. Torbeetti Yeroo 4 fi isaa ol  5.Ji’atti yerooo tokko  6.Kan biroo ( ibsi) | |
| 38 | Fuduraalee baala magariisaa qaban ni nyaattaa(Salaaxaa, goommana)? | 1.Eyyee  2.Lakki | |
| 39 | Yoo deebiin gaaffii # 38 eyyee ta’e, fuduraalee Yeroo ammaamii keessatti nyaatta (Salaaxaa, goommana) ? | 1.Guyyaa guyyaatti  2.Torbeetti Yeroo tokko  3.Torbeetti Yeroo 2-3tti  4. Torbeetti Yeroo 4 fi isaa ol  5.Ji’atti Yeroo tokko  6.kan biroo (ibsi) | |
| 40 | Kuduraalee ni nyaattaa (muuzii, maangoo, burtukaana, paapayyaa? | 1.Yes  2.No | |
| 41 | Yoo kuduraalee ni nyaatta ta’e, yeroo meeqa nyaattaa (muuzii, maangoo, burtukaana, paapayyaa)? | 1.Guyyaa guyyaatti  2.Torbeetti Yeroo tokko  3.Torbeetti Yeroo 2-3tti  4. Torbeetti Yeroo 4 fi isaa ol  5.Ji’atti Yeroo tokko  6.kan biroo (ibsi)---------- | |
| 42 | Bu’aalee aannan irraa argaman ni nyaattaa(dhadhaa, baaduu, itittuu)? | 1. Eyyee  2. Lakki | |
| 43 | Yoo deebiinkee #42 eyyee ta’e, Yoom nyaatta? | 1.Guyyaa guyyaatti  2.Torbeetti Yeroo tokko  3.Torbeetti Yeroo 2-3tti  4. Torbeetti Yeroo 4 fi isaa ol  5.Ji’atti Yeroo tokko  6.kan biroo (ibsi) | |
| 44 | Yoo deebiin gaaffii # 28 eyyee ta’e, bu’aalee aannanii waliin ni fudhattaa ? | 1.Eyyee  2.Lakki | |
| 45 | Shayee ni dhugdaa? | 1. Eyyee  2. Lakki | |
| 46 | Yoo deebiin gaaffii # 45eyyee ta’e, guyyaatti yeroo meeqa? | 1. Yeroo tokko  2. Yeroo 2-3  3. Yeroo 4-5  4. Yeroo 5 ol | |
| 47 | Buna ni dhugdaa ? | 1.Eyyee  2.Lakki | |
| 48 | Yoo deebiin gaaffii # 47 eyyee ta’e, yoom dhugda? | 1.Sa’aa 2 nyaata dura  2. Nyaata waliin  3. Sa’aa 2 nyaata booda  4. Kan biroo( ibsi)_______ | |
| 49 | Nyaata dheedhii ni nyaattaa? | 1.Eyyee  2. Lakki | |
| 50 | Deebiin gaaffii # 49 eyyee yoo ta’e, nyaata dheedhii yoom nyaattaa? | 1.Guyyaa guyyaatti  2.Torbeetti Yeroo tokko  3.Torbeetti Yeroo 2-3tti  4. Torbeetti Yeroo 4 fi isaa ol  5.Ji’atti Yeroo tokko  6.kan biroo (ibsi) | |
| 51 | Yoo deebiin gaaffii # 49 eyyee ta’e, gosoota nyaata dheedhii fi isaan irraa hojjetaman ati nyaattu maalfaadhaa ? | 1. Atarii  2. Shumburaa  3. Talbaa  4.Missira  5.Baaqelaa  6.Nuugii  7.Saliixii  8. Kan biroo (ibsi) --------------- | |
| 52 | Nyaata callaa yookaan nyaata callaarraa hojjetaman ni nyaattaa? | 1.Eyyee  2. Lakki | |
| 53 | Yoo deebiin gaaffii # 52 eyyee ta’e, nyaata callaa irraa hojjetamu yoom? | 1.Guyyaa guyyaatti  2.Torbeetti Yeroo tokko  3.Torbeetti Yeroo 2-3tti  4. Torbeetti Yeroo 4 fi isaa ol  5.Ji’atti Yeroo tokko  6.kan biroo (ibsi)------------ | |
| 54 | Yoo deebiin gaaffii # 52 eyyee ta’e, gosni nyaataa callaa yookaan callaarraa hojjetamanii nyaattu maal faadha? | 1. Daagujjaa  2. Garbuu  3. Qamadii  4. Boqqolloo  5. Ruuzii  6. Paastaa  7. Mukuroonii  8.Xaafii  9. Kan biroo(ibsi)---------------- | |
| 55 | Nyaata yeroo baay’ee nyaattu kamii? | 1. Xaafii  2. Garbuu  3. Qamadii  4. Boqqolloo  5. Daagujjaa  6.Kan biroo (ibsi)----------------- | |
| 56 | Barnoota fayyaa barattee beektaa? | 1. Eyyee  2. Lakki | |
| **IV. Sababoota Dhukkuba Waliin Wal-qabatan** | | | |
| 57 | Dhibee busaatiif ulfa kan ammaarratti yaalamteetta? | 1. Eyyee  2. Lakki | |
| 58 | Saaphana farra bookee busaa siree yeroo ammaa kana ni fayyadamtaa ? | 1.Eyyee  2.Lakki | |
| 59 | Manaa yeroo gadi baatu yeroo hundaa caammaa ni godhataa? | 1. Eyyee  2.Lakki | |
| 60 | Torbee 2’n darban keessatti garaa siteessiseera? | 1.Eyyee  2. Lakki | |
| 61 | Yeroo ammaa kana dhukkuba qabdaa? | 1.Eyyee  2. Lakki | |
| 62 | Deebiin gaaffii # 61 eyyee yoo ta’e, gosni dhukkubakee maalii? | 1. Dhukkuba garaachaa  2.Tiibii  3.HIV/AIDS  4.Dhukkuba Sukkaaraa  5. Kan biroo (ibsi)---------- | |
| **V. Sababoota Qulqullina Waliin wal-qabatan** | | | |
| 63 | Mana fincaaniitti ni fayyadamtaa? | 1.Eyyee  2.Lakki | |
| 64 | Yoo deebiin lakkoofsa 63 eyyee ta’e, bakka harka dhiqannaa qabaa? | 1.Eyyee  2. Lakki | |
| 65 | Yoo deebiin # 63 lakki ta’e, eessatti fayyadamtaa? | 1. Dirreerratti  2.mana fincaanii uummataatti  3.Kan biroo(ibsi)----------- | |
| 66 | Balfa jajjaboo eessatti dhabamsiiftuu? | 1.Dirreerratti  2. Bol’a banaatti  3.Lafa kosiidhaan akka guutamuuf qophaa’etti  4.Kan biroo (ibsi)----------------- | |
| 67 | Balfa dhangala’oo eessatti dhabamsiiftuu? | 1.Dirreerratti  2.Bol’aa banaatti  3. Bol’a qadaadamaatti  4.Kan biroo (ibsi)------------------- | |
| 68 | Maddi bishaan dhugaatii keessanii maalii? | 1. Bishaan bambaa  2. Burqaa yookaan bool’a eegamaa  3. Burqaa hin eegamne  4. Laga  5.Bishaan kuufamaa  6.Kan biroo( ibsi)-------------- | |
| 69 | Maddi bishaan dhugaatii keessanii bambaa miti yoo ta’e ni yaalamaa ? | 1.Eyyee  2. Lakki | |
| 70 | Yoo deebiin gaaffii lakkoofsa 69 eyyee ta’e, mala kamiin fayyadamtuu? | 1. Keemikaala  2. Danfisuu  3. Calaluu  4. Kan biroo (ibsi)------------ | |
| 71 | Nyaata dura yeroo hundaa harka ni dhiqattaa? | 1. Eyyee  2. Lakki | |

**VI. Qabxiilee Gosa Nyaata Haadhaa Ulfaa kan Sa’aa 24 Darbee**

| Lakk | Gareewwan Gosa Nyaataa xiqqaa Haadhaa | Deebii |
| --- | --- | --- |
| 1 | Nyaata istaarchii (Qamadii, Garbuu,Boqqolloo, Ruuzii, Dinnicha) | 1. Eyyee  2.Lakki |
| 2 | Baaqelaa fi Atarii | 1.Eyyee  2. Lakki |
| 3 | Loozii and midhaan sanyii | 1. Eyyee  2.Lakki |
| 4 | Bu’aalee aannanii (dhadhaa, baaduu, itittuu) | 1. Eyyee  2. Lakki |
| 5 | Nyaata foonii | 1. Eyyee  2. Lakki |
| 6 | Hanqaaquu | 1. Eyyee  2. Lakki |
| 7 | Fuduraalee magariisa vitaaminii A of keessaa qabanii (goommana gosa adda addaa) | 1. Eyyee  2. Lakki |
| 8 | Kuduraalee Vitaaminii A of keessaa qabanii (muuzii,,appilii, burtukaana keelloo) | 1. Eyyee  2. Lakki |
| 9 | Fuduraalee biro(dinnicha sukaaraa, kaarotii, saliixa) | 1. Eyyee  2. Lakki |
| 10 | Kuduraalee biroo(Burtukaana, timaatima, qaaraa magariisa) | 1. Eyyee  2.Lakki |

**VII. Bu’aa Laaboraatoriii fi kanneen biroo**

| 1 | Hemoogiloobinii haadhaa | ………g/dl |
| --- | --- | --- |
| 2 | Qorannoo bobbaa | 1 .Raammoon hin jiru  2. Gosa raammoo………..  3. Gosa protozohaa biroo----------------------------------- |
| 3 | Qorannoo Busaa | 1. Hin jiru  2. Jira (gosa)……………… |
| 4 | Qorannoo Fanxoo | A. Jira  B. Hin jiru |
| 5 | Safara irree harka haadhaa (MUAC) | …………….cm |
| 6 | Hojjaa haadhaa | ………………cm |

## Galatoomaa !
