# Supplementary material for: Determinants of anemia among pregnant women attending antenatal care in Horo Guduru Wollega Zone, West Ethiopia: Unmatched case-control study
Source: PLoS One. 2019 Oct 31;14(10):e0224514. doi: 10.1371/journal.pone.0224514 (PMC6822753; doi:10.1371/journal.pone.0224514)
Supplement: Attachment — Submitted filename: 7. Rebutal letter.docx [file pone.0224514.s003.docx]

**Dear editor and reviewers**

PONE-D-19-15457
Determinants of Anemia among Pregnant Women Attending Antenatal Care in Horo Guduru Wollega Zone, West Ethiopia: Unmatched case-control study

First of all, we would like to thank you for providing the reviewers’ comments which are very constructive and crucial. We have incorporated all the point by point below. We have formatted the manuscript based on the PLOSONE guidelines. We have edited the English language online since we cannot afford to pay for editing agents. We have cleaned the manuscript as well. We have marked “red” the changes made in the manuscript and we have a separate file for “manuscript with track changes” and “clean manuscript”. We have also attached a “rebuttal letter” which responds editor and reviewers’ comments and/suggestions point by point. Further another document is also added as additional material to minimize the number of tables.

Top of Form

**View LetterResponse to editor**

1. We have included literatures from abroad and thus our manuscript currently does not look a replication of previous works done in Ethiopia.
2. We have copyedit manuscript for language usage, spelling, and grammar. The language in the manuscript looks better than it was before
3. We have included additional information regarding the survey or questionnaire used in the study and we ensure that we have provided sufficient details that others could replicate the analyses. The validation and pre-test regarding the questionnaire is included in the manuscript.
4. We have corrected the P value reference to "p<0.001"
5. We have changed our declaration regarding data availability to “All data and supporting documents are available freely without restriction”. We have added questionnaires used freely.

**Response to the reviewers**

1. Women were recruited at ANC visit. The inclusion criteria were unclear especially regarding which ANC visit. What was the gestational age at inclusion? How was gestational age assessed? These are important criterias to mention and to discuss: Hb changes over the course of pregnancy (Ouedraogo et al.). Different cut-offs have been proposed according to the timing in pregnancy. This is not explained nor discussed, although this introduces some change in women included or not in the case/control group.

**Response**: we have included clear inclusion criteria in the manuscript. The gestational age was assessed by the participants’ record books and patients’ response. The women were recruited in any ANC visit with any gestational age. Anemia definition cut-off value was categorized based on gestational age in both cases and controls as clearly described in the manuscript.

2. Also, what are guidelines in Ethiopia regarding anemia, malaria, iron/folic acid supplements, treatments for helminths, and their timing of administration? That would influence the level of anemia according to gestational age, and risk factors according to gestational age.

**Response**: We used WHO and Ethiopian ANC follow up guideline to define anemia in pregnant women Factors like malaria, iron/Folic acid supplements and deworming prophylaxis administration status those included on anemia guideline in Ethiopia were incorporated in the study.We have used Ethiopian ANC follow-up guideline to analyze our data regarding anemia and other factors

3. Some parts are too detailed for a scientific article. For example, data quality control, SOPs, etc. As well as some parts of data analysis. 100% response rate: this is strange. All women accepted to participate? Severity of anemia should be described. The language should be revised.

**Response**: We have briefly summarized the detailed parts like data quality control and SOPs and the data analysis. The response rate was 100% since we used consecutive sampling technique. We enrolled pregnant women consecutively until the sample size was reached. Thus we have removed the phrase “100% response rate”. Severity of anemia was described as Mild, moderate and severe in the manuscript. We have edited the language well.

4. The results should refer to the tables explicitly. The p-value should be added in the tables were appropriate. Some abbreviations in the tables are not explained (for example, MUAC, DDS).

**Response**: The results refer the tables clearly. All P-values were added in the tables where appropriate and all abbreviations are explained.

5. Intestinal helminthic infections and the type of helminths infection are both included in the multivariate analyses, as presented. However, they are highly correlated, and it makes no sense to include both of them. The number of tables should be reduced.

**Response**: We have removed the “intestinal helminthic infection” from the multivariate analysis. We have reduced the number of tables from 6 to 4 tables. We separately submitted the removed tables as “Supporting Table ST1”

Bottom of Form

 Close

OKCancel

ErrorClose

OK

 Close

OK
