# Supplementary material for: Determinants of anemia among pregnant women attending antenatal care in Horo Guduru Wollega Zone, West Ethiopia: Unmatched case-control study
Source: PLoS One. 2019 Oct 31;14(10):e0224514. doi: 10.1371/journal.pone.0224514 (PMC6822753; doi:10.1371/journal.pone.0224514)
Supplement: Attachment — Submitted filename: 6. Response to reviewers.docx [file pone.0224514.s004.docx]

**Dear editor and reviewers**

PONE-D-19-15457R1
Determinants of Anemia among Pregnant Women Attending Antenatal Care in Horo Guduru Wollega Zone, West Ethiopia: Unmatched case-control study

First of all, we would like to thank you for providing the reviewers’ comments which are very constructive and crucial. We have incorporated all the point by point below. We have highlighted “red” the changes made in the manuscript and we have a separate file for “manuscript with track changes” and a clean manuscript “manuscript”. We have also attached a rebuttal letter “Response to reviewers” which responds reviewers’ comments and/suggestions point by point.

**Response to the reviewers**

1. There is still a major comment which is gestational age at enrolment. Gestational age at enrolment should be described. Indeed, the definition of anemia changes over pregnancy, and iron supplementts, anti-helminthic treatments, IPTp, etc are prescribed to the women during pregnancy. Thus, depending on the timing during pregnancy of enrolment in the study, risk factors may change. This should be discussed in the manuscript.

**Response:** Pregnant women at any gestational age (GA) were included in the study. GA of pregnant women was classified into trimesters i.e. GA below 12 weeks: first trimester, GA of 13- 24 weeks: second trimester and GA above 24 weeks: third trimester. The gestational age was calculated from last normal menstrual period (LNMP) which was determined by antenatal care providers. In Ethiopia, health extension workers register pregnant women of any GA with their last normal menstrual period at home, thus, GA is not usually missed. The proportion of gestational age among the study participants is already described in the manuscript and supporting files.

GA was not a significant predictor of anemia among cases and multicollinearity test was done by using VIF to assess presence of collinearity among independent variables and the value of VIF was within the acceptable range which showed no collinearity. Therefore, there was no correlation between GA and other risk factors of anemia in our study which means the risk factors of anemia were not changeable based on differences in gestational age.

Bottom of Form

 Close

OKCancel

ErrorClose

OK

 Close

OK
